# Physician's perceptions regarding the pharmaceutical industry: a Brazilian national study

**DOI:** 10.1590/1806-9282.20231317

**Published:** 2024-05-20

**Authors:** Giovana Rosa Gameiro, Gustavo Rosa Gameiro, Renan Magalhães e Silva, Aline Gil Alves Guilloux, Alex Jones Flores Cassenote, Mario César Scheffer

**Affiliations:** 1 Clínica de Olhos Norte do Paraná – Londrina (PR), Brazil.; 2 Universidade de São Paulo, Faculty of Medicine, Medical Education Development Center – São Paulo (SP), Brazil.; 3 Universidade Federal de São Paulo, Paulista School of Medicine, Department of Ophthalmology and Visual Sciences – São Paulo (SP), Brazil.; 4 Universidade de São Paulo, Faculty of Medicine, Department of Preventive Medicine – São Paulo (SP), Brazil.

**Keywords:** Medical education, Pharmaceutical industry, Physicians, Medical ethics, Conflict of interest

## Abstract

**OBJECTIVE:**

The objective of this study was to investigate the newly graduated physicians' attitudes and perceptions regarding the medical relationship with the pharmaceutical industry and identify the sociodemographic patterns related to such thinking.

**METHODS:**

A structured questionnaire was administered to 4,601 participants selected from a pool of 16,323 physicians who were registered with one of the 27 Regional Medical Councils of Brazil in 2015. Answers were analyzed using two stratification variables: type of medical school (public vs. private) and the sex of the respondents.

**RESULTS:**

Out of the participants, 61.8% believed that industry funding could support medical conferences and education, and 48.4% felt that small gifts and conference travel funding were acceptable. Conversely, 64.7% disagreed with industry-sponsored social events. Views on whether pharmaceutical representatives' visits influenced prescriptions were divided. Statistically significant differences were observed between genders and medical school types, with men and private school graduates being more accepting of certain industry interactions.

**CONCLUSION:**

The study highlights the nuanced attitudes of new doctors toward industry relationships, indicating the need for clearer ethical guidelines and education in medical schools to align practice with evolving societal values.

## INTRODUCTION

In recent years, in the light of progressively fewer boundaries between medicine and the pharmaceutical industry, there has been increasing concern regarding the interactions between the drug industry and physiciansg^1,2^. Studies from many countries show that exposure to drug companies influences physicians' prescription choices and may affect evidence-based medical practice, prescribing costs, and patient safety^2,3^.

Despite the rapidly evolving pharmaceutical industry in developing countries and the great impact that their marketing strategies and interactions with physicians may have on medical practice^4^, literature assessing the doctor–industry relationship in Brazil is quite sparse^5,6^.

To the best of our knowledge, this is the first Brazilian study with national proportions not only to investigate the newly graduated physicians' attitudes and perceptions regarding the medical relationship with the pharmaceutical industry but also to identify the sociodemographic patterns related to such thinking.

## METHODS

This paper is part of the research "Profile and Perceptions of New Graduates in Brazil," a 104-structured multiple-choice questionnaire grouped into 11 thematic groups aimed at addressing the demographic profile of new qualified physicians registered with one of the 27 Regional Boards of Medicine (CRMs) in Brazil. This survey was conducted between September 2014 and August 2015. This study focuses on the relationship between the pharmaceutical industry and recently graduated doctors, and it builds upon prior investigations conducted utilizing the same survey tool. Among the established questions, 12 were related to the socioeconomic conditions of each participant and 5 were linked to the viewpoints of the graduates regarding the pharmaceutical industry.

The study involved 4,601 volunteer participants from a pool of 16,203 recent medical school graduates. The process of survey development, inclusion and exclusion guidelines, validation procedures, and distribution methods have been detailed in previous publications^5-7^.

The sampling process included the use of the following stratification variables: the type of medical school, the sex of the respondents, and the Brazilian region of the medical school. The number of participants varied across questions and within each subgroup. A stratified sampling approach was used to correct the representativeness of these subgroups in the population results, avoiding the bias that could arise from the voluntary adherence sampling method. The correction factor was defined by the fraction of the target population within each subgroup. The 95% confidence intervals for frequencies were computed by bootstrapping. All the analyses were performed in the IBM SPSS Statistics software version 25 (IBM Corp., Armonk, NY)^5-7^.

## RESULTS

A comprehensive exploration of the socio-demographic attributes of the participants was previously delineated by Scheffer et al.^6^, providing an in-depth depiction of the graduates' profiles. The opinions of the fresh medical graduates regarding the medical relationship with the pharmaceutical industry are summarized in Table 1.

**Table 1:** Fresh graduate physicians' opinions about the medical relationship with the pharmaceutical industry.

	I agree			Disagree			I prefer not to answer		
	n	Freq. %	95%CI	n	Freq. %	95%CI	n	Freq. %	95%CI
Medical conferences, publications, and continuing education programs can be financed by the industry.	1,988	61.8	58.5–65.0	724	21.5	19.3–23.8	527	16.7	14.8–18.9
The doctor can receive gifts of small value and travel funding for conferences.	1,559	48.4	44.2–52.6	1,063	32	29.6–34.5	614	19.6	16.9–22.5
The industry representative visit influences the doctor's prescription practices.	1,447	42.6	38.1–47.2	1,391	44.2	41.1–47.4	397	13.2	11.7–14.9
It is correct for the industry to finance "cervejadas," barbecues, and cocktails for students and residents.	525	16.2	12.6–20.6	2,094	64.7	60.0–69.2	617	19.1	17.2–21.2
The physician should be prohibited from linking medical prescription practices to the receipt of material benefits or financial support.	2,369	72.9	70.4–75.2	385	11.6	10.4–12.9	478	15.5	13.6–17.7

The percentages were obtained through weighing of individuals, so the direct division of cells by the totals in this table will yield incorrect results and therefore are discouraged.

The physicians' relationship with the pharmaceutical industry was a controversial topic among medical graduates. Among them, 61.8% agreed that medical conferences, publications, and continuing medical education programs can be financed by the industry, and 48.4% agreed that "the doctor can receive small gifts and conference travel funding." Almost 65% disagreed that it is correct for the industry to sponsor parties, barbecues, and cocktails for students and residents; however, 16.2% of respondents believe that this practice is correct. The graduates were divided regarding the statement "the visit of the representative influences the doctor's prescription"—42.6% of respondents agreed with the statement, while 44.2% disagreed. Approximately 73% judged that the physician should be prohibited from linking a medical prescription to the receipt of material advantages or financial support, although 11.6% agreed with this practice.

The participants' judgment about the relationship with the pharmaceutical industry varied significantly according to the variables sex (Table 2) and type of medical school (Table 3). Men agreed more often than women regarding the ethical adequacy of industry-funded publications and continuing medical education programs (66.0 vs. 59.3%, p=0.019), receiving small gifts and conference travel funding (53.9 vs. 45%, p=0.037), and having pharmaceutical-company sponsored parties, barbecues, and cocktails (21.6 vs. 12.9%, p=0.002). Public school graduates more often consider that the visit of industry representatives influences the doctor's prescription than private school graduates (51.5 vs. 39.5%), who in turn disagree more with this statement (46.3 vs. 38.2%), p<0.001.

**Table 2:** Opinions of fresh graduates on the relationship between physicians and the pharmaceutical industry significantly differences according to gender.

		Gender								
		Male			Female			Total		
		Freq. %	95%CI	n	Freq. %	95%CI	n	Freq. %	95%CI	n
1Medical conferences, publications, and continuing education programs can be financed by the industry.										
	I agree	66	64.8–67.8	961	59.3	57.8–60.8	1,027	61.4	58.5–65.0	884
	Disagree	19.5	16.1–23.4	307	22.7	20.9–24.5	417	22.4	19.3–23.8	2,023
	I prefer not to answer	14.5	12.7–17.2	210	18.1	17.0–19.2	317	16.3	14.8–18.9	770
2The doctor can receive gifts of small value and travel funding for conferences.										
	I agree	53.9	52.3–55.6	778	45	43.4–46.7	781	48.4	44.2–52.6	1,559
	Disagree	29.9	25.9–34.2	466	33.3	31.9–34.8	597	32	29.6–34.5	1,063
	I prefer not to answer	16.2	13.4–19.4	235	21.6	20.4–23.0	379	19.6	16.9–22.5	614
3It is correct for the industry to finance "cervejadas," barbecues, and cocktails for students and residents.										
	I agree	21.6	17.7–26.2	317	12.9	11.4–14.5	208	16.2	12.6–20.6	525
	Disagree	59.8	56.4–63.0	905	67.7	66.2–69.2	1,189	64.7	60.0–69.2	2,094
	I prefer not to answer	18.6	14.1–24.2	256	19.4	17.8–21.2	361	19.1	17.2–21.2	617

1 p=0.019

2 p=0.037

3 p=0.002.

**Table 3:** Fresh graduate physicians' significantly different opinions on the relationship between physicians and the pharmaceutical industry stratified by the type of medical school.

		Type of medical school								
		Public			Private			Total		
		Freq. %	95%CI	n	Freq. %	95%CI	n	Freq. %	95%CI	n
1The doctor can receive gifts of small value and travel funding for conferences.										
	I agree	50.3	47.0–53.6	725	47.7	42.4–53.1	834	48.4	44.2–52.6	1,559
	Disagree	34.4	33.3–35.6	498	31.2	28.2–34.3	565	32	29.6–34.5	1,063
	I prefer not to answer	15.3	12.1–19.1	226	21.1	18.8–23.5	388	19.6	16.9–22.5	614
2The industry representative visit influences the doctor's prescription practices.										
	I agree	51.5	48.8–54.1	728	39.5	38.4–40.7	719	42.6	38.1–47.2	1,447
	Disagree	38.2	35.2–41.3	570	46.3	45.4–47.1	821	44.2	41.1–47.4	1,391
	I prefer not to answer	10.3	8.8–12.1	150	14.2	13.6–14.9	247	13.2	11.7–14.9	397
2The physician should be prohibited from linking medical prescription practices to the receipt of material benefits or financial support.										
	I agree	77.9	75.2–80.4	1,110	71.1	69.3–72.9	1,259	72.9	70.4–75.2	2,369
	Disagree	10.9	9.2–12.8	166	11.8	10.3–13.6	219	11.6	10.4–12.9	385
	I prefer not to answer	11.2	9.6–13.1	171	17	16.6–17.5	307	15.5	13.6–17.7	478

1 p=0.037

2 p<0.001.

## DISCUSSION

It is widely known that the pharmaceutical industry has a huge economic impact worldwide, generating billions of dollars in revenue annually^1,8^. As part of the strategy to further increase their sales and profit, a considerable amount of money is spent on marketing to physicians, which includes pharmaceutical sales representative visits, sponsorship of conferences and other continuing medical education programs, drug promotional offers, free samples, and gifts^1,4^.

Research has shown that physician–industry interactions may result in a prescribing behavior that deviates from evidence-based guidelines and, therefore, from the patient's best interest and safety^1-3^. This can be exemplified in the prescription of drugs without clear benefits over the other options, the request for more expensive drugs, the decrease in the use of generic drugs, and the prescription of medications based on the availability of drug samples or in the relationship with pharmaceutical representatives^1-3^. It has been exposed that the frequency of visits to a physician by industry representatives is linked to an increase in the physician's inclination to prescribe the representative's product^9^. Moreover, the non-rational prescribing behavior can also affect patient trust in physicians^3^.

Considering the current scenario in which over 50% of the medical consultations result in drug prescription^10^, and in the light of the previously cited negative influence of drug company marketing strategies on physicians' prescription choices^1,2,4^, many medical organizations worldwide, such as the American Medical Association and the American Medical Student Association, have developed recommendations toward the interaction between physicians or medical students and the industry^4^.

In Brazil, there is no specific legislation on this topic, unlike in the United States, which has the "Sunshine Act"^11^. Notwithstanding, there are some ongoing legislative projects in the Chamber of Deputies^12-15^. The Code of Medical Ethics in Brazil states that doctors are prohibited from practicing medicine with ties to or dependence on industries of any nature^16^. Additionally, the Association of Research-Based Pharmaceutical Industry in Brazil also has a code of conduct that discusses aspects of the medical–industry relationship, with the aim to guide ethical decisions and promote a culture of compliance^17^.

In the literature, physician exposure to drug companies was found to be widespread worldwide. Previous studies from Brazil^8,10,18^ and other countries such as Turkey^2^, Japan^3^, the United States^1^, and Germany^19^ have shown that doctors and medical students frequently interact with the pharmaceutical industry, and a high percentage of them report having received small gifts, having attended drug company-sponsored events or meals, and even having scientific publication fees sponsored^18^.

A percentage of 61.8% of respondents believe the industry can sponsor medical conferences, publications, and continuing medical education. However, the literature presents conflicting results. In a previous Brazilian study, medical students viewed industry funding for conferences, research, and publications as potentially unethical^8^. On the contrary, in a study from the United States, 89% of medical students agreed that most industry-sponsored grand rounds are helpful and educational, and only 11% disagreed.^1^ A Pakistani study also showed that 81% of medical students supported pharmaceutical sponsorship of educational events^4^, and a Japanese study echoed this positive sentiment toward industry-backed seminars^3^.

Our study found that 48.4% of participants believed that doctors could accept minor gifts and conference travel funding from the industry. This sentiment aligns with global findings. For instance, in the United States, over 80% of medical students felt entitled to gifts from drug companies, with nearly 70% believing these would not influence their practices^1^. Similarly, a Japanese survey revealed that 67% of medical students saw no issue with small gifts, though only 10% believed that such gifts or meals could sway their practices^3^. A German study supported these views, with 45.6% finding minor gifts acceptable due to their perceived minimal impact and 25% considering them influential on prescribing behavior^19^.

In developing nations, medical students often hold divided opinions about accepting industry gifts. A Pakistani study found over 40% of the students were neutral regarding considering it unacceptable for a physician to receive a gift from a drug company in any form, with approximately 30% considering it acceptable and 27% considering it unacceptable^4^. Similarly, a Turkish study showed that 33% believed medical students should always decline industry gifts^2^. In the literature, it has been shown that receiving a gift or compensation may alter the physician's attitude toward the person who gave the gift^9^.

This study revealed that 65% of participants disagreed with industry-sponsored social gatherings for students and residents, while 16.2% supported it. A higher acceptance rate was found in a US. study, in which 30% of the medical students considered it appropriate and 31.6% were neutral regarding social outings being sponsored by drug companies. Considering industry-funded meals, 77% found it appropriate^1^. In Japan, only around 9% of medical students in clinical years considered it inappropriate to have industry-sponsored meals, with 40.4 and 25.5% totally agreeing and somewhat agreeing with it, respectively. Additionally, 65% of the students believed industry-sponsored lunches would not impact their clinical practice^3^.

Regarding the influence of pharmaceutical representative visits on prescription writing, 42.6% of the respondents agreed that doctors are influenced by it, and 44.2% disagreed. These results are supported by a previous Brazilian study, which found mixed perceptions among medical students—43.2% felt unaffected by representative visits, while 42% agreed that doctors are often influenced^10^. Another Brazilian study also highlighted the belief that industry marketing strategies could affect prescription writing^8^.

International studies from the United States^1^, Germany^19^, and Pakistan^4^ indicated that only a minority of medical students expected their future prescriptions to be influenced by pharmaceutical gifts or incentives. In contrast, a Turkish study found about 70% of final-year students believed drug company interactions impact physicians' prescribing preferences^2^. Interestingly, past research revealed a common belief among medical students and doctors that colleagues are more susceptible to industry influence than themselves^1,10,19^.

Our findings exposed that 72.9% of the respondents believe that physicians should not receive benefits or compensation for prescribing specific drugs, aligning with the Brazilian Code of Medical Ethics^16^. However, 11.6% of the respondents disagreed with it, expressing an opinion contrary to the current ethical regulation. A Pakistani study yielded similar results: 70% of medical students oppose doctors receiving financial incentives from drug companies for prescriptions^4^.

Considering that the first interaction between medical students and drug companies starts early in their training in medical school, during a time that shapes their professional conduct and future prescribing behavior^2,4,8^, it would be crucial for the universities and academic regulatory agencies to implement policies regulating the medical student–pharmaceutical industry interaction^2,4,18^. Future medical school curriculum reforms to include wide discussion and formal guidance on the topic, as well as courses that stimulate rational prescribing behaviors and evidence-based medicine and that reinforce conflict of interest policies would also be beneficial^2,4,18^.

As medical students also learn from the attitudes and examples set by physicians, doctors who work in medical schools should also receive training^18^. Ensuring continuous monitoring and adaptation of regulations are important steps in promoting ethical conduct and professionalism in the relationship between physicians and the pharmaceutical industry.

Furthermore, this study revealed statistically significant gender-based disparities in views on the physician–industry relationship: men were more favorable than women toward industry sponsorship of publications, medical education programs, gifts, parties, cocktails, and travel funding. In the literature, no previous Brazilian study had analyzed this variable. In contrast, a Japanese study found no significant gender differences among medical students in their stance on accepting gifts from the pharmaceutical industry^3^. Future studies may explore possible variations in behaviors and ethical-professional attitudes according to gender but should also consider that certain types of practice and medical specialties, predominantly male, may be more exposed to direct relationships with the industry.

Our study revealed significant disparities based on the legal status of participants' medical schools. Public school graduates in Brazil more often believed that industry visits affect prescriptions (51.5 vs. 39.5% for private school graduates). This is the first Brazilian study delving into this, with no previous national research comparing physician or student interactions with the pharmaceutical industry in private versus public settings. A Pakistani study found that private school students were more skeptical of pharmaceutical company information and more comfortable accepting expensive gifts^4^. A Japanese survey also noted private school students were more receptive to accepting textbooks and sponsored lunches from drug firms than their public school peers^3^.

Our results might reflect the different levels of exposure to pharmaceutical marketing between the private and public settings and possible differences in the medical school curriculum of private and public schools. The socioeconomic backgrounds of students might also contribute to this outcome^4^. However, it is noteworthy that the opinion on physician–industry relationship was not significantly associated with family income in our study. Participants with family income greater or equal to 10 minimum wages had similar opinions to those with less than 10 minimum wages.

## LIMITATIONS

Despite encompassing a significant participant pool from all regions in Brazil, this study has some limitations. As a cross-sectional study, it lacks temporal insights regarding changes in student's opinions over time, and as we can only measure correlations, conclusions regarding causal relationships cannot be reached. Furthermore, there is an 8-year interval between data collection and publication of the results. However, it is useful to register these findings in the literature to enable comparisons with future studies. Further prospective or intervention studies involving medical students and doctors at different career stages and from different specialties are suggested for a more comprehensive understanding of the factors affecting their opinions toward the pharmaceutical industry. This could help institutions frame ethical policies on physician–industry interactions.

## CONCLUSION

Despite the presence of regulations and medical ethical codes serving as benchmarks to delineate boundaries to industry operation, our study indicates the level of permissiveness recent graduates might exhibit toward gifts and incentives from this industry. This demonstrates that while laws might reflect the change in social values and norms over time, they do not necessarily reflect the totality of people's attitudes and personal opinions.

Universities may consider implementing policies regulating medical student interactions with pharmaceutical companies and incorporate training in evidence-based medicine and conflict of interest into the curriculum, beginning in the first years of medical school, which is when the first connection between medical students and the industry usually occurs, shaping their professional demeanor and future prescribing habits. Continuous education for medical school physicians—who act as role models—and regular policy updates are also crucial to maintain professionalism in the industry relationship. New investigations should be carried out, perhaps even with qualitative studies, as well as a more focused approach in medical schools regarding the effectiveness of new processes and curriculum modifications.

## Data Availability

The datasets used and analyzed during this study are available from the corresponding author upon reasonable request.
